# Impact of the COVID-19 pandemic on publication dynamics and non-COVID-19 research production

**DOI:** 10.1186/s12874-021-01404-9

**Published:** 2021-11-22

**Authors:** Marc Raynaud, Valentin Goutaudier, Kevin Louis, Solaf Al-Awadhi, Quentin Dubourg, Agathe Truchot, Romain Brousse, Nouredine Saleh, Alessia Giarraputo, Charlotte Debiais, Zeynep Demir, Anaïs Certain, Francine Tacafred, Esteban Cortes-Garcia, Safia Yanes, Jessy Dagobert, Sofia Naser, Blaise Robin, Élodie Bailly, Xavier Jouven, Peter P. Reese, Alexandre Loupy

**Affiliations:** 1 Paris Translational Research Epidemiology and Biostatistics Department, Université de Paris, INSERM U970, PARCC, 56 rue Leblanc, 75015 Paris, France; 2 Pitié-Salpêtrière University Hospital, Assistance Publique-Hôpitaux de Paris, Sorbonne University, Paris, France; 3 Kidney Transplantation Department, Necker Hospital, Assistance Publique – Hôpitaux de Paris, Paris, France; 4 Paediatrics Unit, Necker University Hospital, Paris, France; 5 Nephrology, Dialysis and Transplantation Department, Hospital Privado Universitario de Cordoba, Cordoba, Argentina; 6 Thomas E. Starzl Transplantation Institute, University of Pittsburgh School of Medicine, Pittsburgh, PA USA; 7 Cardiology Departement, Hôpital Européen Georges Pompidou, Paris, France; 8 University of Pennsylvania School of Medicine, Philadelphia, PA USA

**Keywords:** COVID-19, Meta-research, Publications, High-impact journals

## Abstract

**Background:**

The COVID-19 pandemic has severely affected health systems and medical research worldwide but its impact on the global publication dynamics and non-COVID-19 research has not been measured. We hypothesized that the COVID-19 pandemic may have impacted the scientific production of non-COVID-19 research.

**Methods:**

We conducted a comprehensive meta-research on studies (original articles, research letters and case reports) published between 01/01/2019 and 01/01/2021 in 10 high-impact medical and infectious disease journals (New England Journal of Medicine, Lancet, Journal of the American Medical Association, Nature Medicine, British Medical Journal, Annals of Internal Medicine, Lancet Global Health, Lancet Public Health, Lancet Infectious Disease and Clinical Infectious Disease). For each publication, we recorded publication date, publication type, number of authors, whether the publication was related to COVID-19, whether the publication was based on a case series, and the number of patients included in the study if the publication was based on a case report or a case series. We estimated the publication dynamics with a locally estimated scatterplot smoothing method. A Natural Language Processing algorithm was designed to calculate the number of authors for each publication. We simulated the number of non-COVID-19 studies that could have been published during the pandemic by extrapolating the publication dynamics of 2019 to 2020, and comparing the expected number to the observed number of studies.

**Results:**

Among the 22,525 studies assessed, 6319 met the inclusion criteria, of which 1022 (16.2%) were related to COVID-19 research. A dramatic increase in the number of publications in general journals was observed from February to April 2020 from a weekly median number of publications of 4.0 (IQR: 2.8–5.5) to 19.5 (IQR: 15.8–24.8) (*p* < 0.001), followed afterwards by a pattern of stability with a weekly median number of publications of 10.0 (IQR: 6.0–14.0) until December 2020 (*p* = 0.045 in comparison with April). Two prototypical editorial strategies were found: 1) journals that maintained the volume of non-COVID-19 publications while integrating COVID-19 research and thus increased their overall scientific production, and 2) journals that decreased the volume of non-COVID-19 publications while integrating COVID-19 publications. We estimated using simulation models that the COVID pandemic was associated with a 18% decrease in the production of non-COVID-19 research. We also found a significant change of the publication type in COVID-19 research as compared with non-COVID-19 research illustrated by a decrease in the number of original articles, (47.9% in COVID-19 publications vs 71.3% in non-COVID-19 publications, *p* < 0.001). Last, COVID-19 publications showed a higher number of authors, especially for case reports with a median of 9.0 authors (IQR: 6.0–13.0) in COVID-19 publications, compared to a median of 4.0 authors (IQR: 3.0–6.0) in non-COVID-19 publications (*p* < 0.001).

**Conclusion:**

In this meta-research gathering publications from high-impact medical journals, we have shown that the dramatic rise in COVID-19 publications was accompanied by a substantial decrease of non-COVID-19 research.

**Meta-research registration:**

https://osf.io/9vtzp/.

**Supplementary Information:**

The online version contains supplementary material available at 10.1186/s12874-021-01404-9.

## Background

With a total of 3,541,881 deaths among 170,360,315 confirmed cases [1] as of May 31st, 2021, the coronavirus disease 2019 (COVID-19) pandemic has placed a strain on health systems worldwide. According to the Organization for Economic Co-operation and Development, an estimated 7 billion dollars, dedicated to COVID-19 research, were unlocked worldwide in the first 9 months of 2020 [2]. Linked to that, major collaborative efforts have been launched to urgently address COVID-19 related medical issues [3, 4], sometimes at the expense of non-COVID-19 research [5]. Some medical fields have experienced a decrease in funding allocation and publications [6], which had potentially affected patient care outside of COVID-19. For instance, the pandemic has seriously impacted cancer patients with treatment delays and reduced access to healthcare [7]. Similarly, it has had detrimental effects on organ allocation and transplantation worldwide [8], with a significant reduction in the number of transplanted organs per day, with disastrous consequences for patients whose lives depend on getting transplanted.

Overall, there has been a substantial redistribution of resources which has significantly impacted the non-COVID-19 medical research worldwide [9], including clinical trials [10, 11]. In addition, leading scientists have voiced concerns about science expediency [12] and the lowering of scientific standards [13, 14]. Together, these phenomena could have played a significant role on the dynamics of publication and worldwide medical research.

Moreover, recent research has reported a rising number of authors in COVID-19 publications [15], especially in case reports [16, 17], which may also reflect a lowering in scientific standards [12, 13]. In medical science, there has been a constant rise in the number of authors since the 1950s [18]. This phenomenon has been highlighted in numerous medical specialties [19–21] and may be the aggregate consequence of multiple forces, such as the growing complexity [22] and interdisciplinarity of medical research [23], increasing academic and career pressure, increasingly limited funding and the rising number of collaborations [23]. However, the impact of a pandemic on the number of authors has not been investigated.

We made the hypothesis that a worldwide pandemic such as COVID-19 may impact the medical research and in particular non COVID-19 scientific production [15].

Therefore, to address these questions, we conducted a meta-research to comprehensively investigate the effects of the COVID-19 pandemic on the medical research publication dynamics and the impact of COVID-19 research on non-COVID-19 research.

## Methods

### Search strategy

We followed the Preferred Reporting Items for Systematic Reviews and Meta-Analyses (PRISMA) statement to design and report our meta-research, where applicable (see protocol). A literature search of PubMed was performed between January 1st 2019 and January 1st 2021, for articles published in medical, broad journals and journals specializing in infectious disease and public health with an impact factor greater than 8. It hence included the ten following journals: New England Journal of Medicine, Lancet, Journal of the American Medical Association, Nature Medicine, British Medical Journal, Annals of Internal Medicine, Lancet Global Health, Lancet Public Health, Lancet Infectious Disease and Clinical Infectious Disease.

Two researchers (VG, KL) independently implemented the search strategy and did the data extraction to ensure that the same references were identified. The references of the included medical articles and relevant reviews were scanned for potentially relevant medical articles that may have been missed in the literature search. We also requested potentially eligible medical articles from content experts. The search strategy is presented in the supplementary methods with the study protocol, which has been retrospectively registered, at https://osf.io/9vtzp/.

### Inclusion criteria

We included all English-language publications with original data, comprising original articles, research letters (and corresponding synonyms) and case reports. Publications without original data (editorial, perspective, viewpoint, narrative reviews, etc.) were excluded.

### Screening and data extraction

All references were screened according to the titles, abstracts and full texts by 20 reviewers (MR, VG, KL, SA, QD, AT, RB, NS, AG, CD, ZD, MD, SN, EB, BR, AC, JD, SY, ECG, FT). The following data from each article were extracted: (1) basic information: journal, title, publication date, name of first author, (2) publication type, (3) number of authors (for consortia, we considered the total number of authors), (4) whether the publication was COVID-19 related or not, (5) whether the publication was based on case series, (6) number of patients for case reports and case series. Uncertainty in the categorization was resolved through a weekly discussion with all members. After the screening completion, three independent reviewers (MR, VG, KL) randomly checked 20% of the references for each reviewer - except theirs. If more than 3% of inconsistencies were observed for one given reviewer, a re-evaluation with re-adjudication was conducted for all the references of the reviewer.

### Data analysis

#### Publication dynamics

We aimed at estimating the overall trend of the publication dynamics and the associations with the publications type and journals. To do so, we used a locally estimated scatterplot smoothing (LOESS) method using the *smooth* function in R. We represented the weekly number of publications over time, and we used the Wilcoxon test to compare the weekly number of publications between periods of time.

#### Publication type and COVID-19

We aimed at investigating the publication type in COVID-19 studies and non-COVID-19 studies, which are characterized by case report, research letter, original article. The Chi^2^ test was used to assess the difference in proportions of these publication types.

#### Calculation of the number of authors

We aimed at investigating the number of authors and the associations with the type of publications. A Natural Language Processing algorithm was specifically designed to calculate the number of authors for each publication.

#### Number of authors dynamics and impact on medical research

We aimed at estimating the overall trend of the number of authors dynamics and the associations with the publications type. To do so, we used the LOESS method described above. We represented the weekly-estimated, median number of authors for the number of authors dynamics. We further compared the difference in the median number of authors between COVID-19 and non-COVID-19 publications, stratified by publication type, with the Wilcoxon test.

#### Simulation of the number of unpublished non-COVID-19 studies

We aimed at estimating the number of non-COVID-19 studies that could have been published during the pandemic period. First, using the LOESS method described above, we extrapolated the publication dynamics observed from January 1st 2019 to the start of the pandemic which was set on the 30th January 2020, following the official declaration of the World Health Organization [24]. Based on this trend, we then simulated the number of non-COVID-19 studies that could have been published, from 31st January 2020 to 31st December 2020, if the pandemic had not occurred. We then subtracted the number of non-COVID-19 studies that were actually published to obtain the final, simulated number of unpublished non-COVID-19 studies.

All analyses were performed with Endnote (Endnote X9, Thomson Reuters), NoteExpress (Version 3.2, Beijing Aegean Software Co., Ltd.,) and R (version 3.2.1, R Foundation for Statistical Computing) software. Data are available upon reasonable request.

## Results

**Fig. 1:**
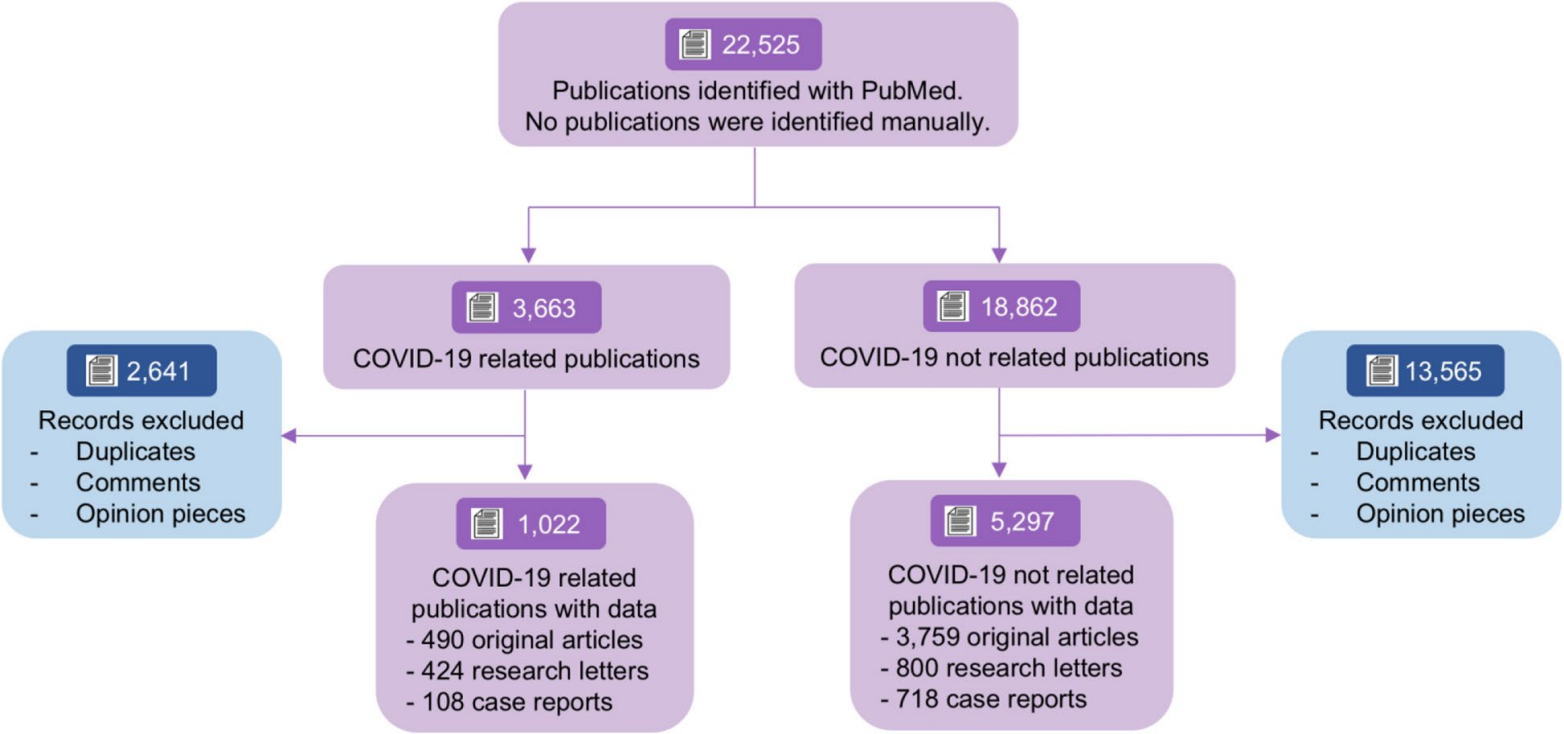
Study flowchart. The flowchart depicts the review process and the inclusion/exclusion criteria. PubMed data source were used for identifying publications from the 10 high-impact medical journals included in the present study (New England Journal of Medicine, Lancet, Journal of the American Medical Association, Nature Medicine, British Medical Journal, Annals of Internal Medicine, Lancet Infectious Disease, Lancet Global Health, Lancet Public Health and Clinical Infectious Disease). We did not retrieve any additional publications with manual search

A total of 22,525 references were identified in the top ten medical journals, of which 3663 (16.3%) were COVID-19 related publications and 18,862 (83.7%) were non-COVID-19 related publications (Fig. 1). After removing duplicates and publications which did not include original data (editorial, perspective, viewpoint, narrative reviews, etc.), 6319 publications with original data remained for the final analyses. One thousand twenty-two were related to COVID-19 (16.2%), and 5297 (83.8%) were not.

### Impact of the COVID-19 pandemic on the publication dynamics

#### Overall publication dynamics

In the year 2020, COVID-19 publications accounted for 1022 (25.9%) of the total number of publications while the non-COVID-19 ones accounted for 2930 publications (74.1%).

**Fig. 2:**
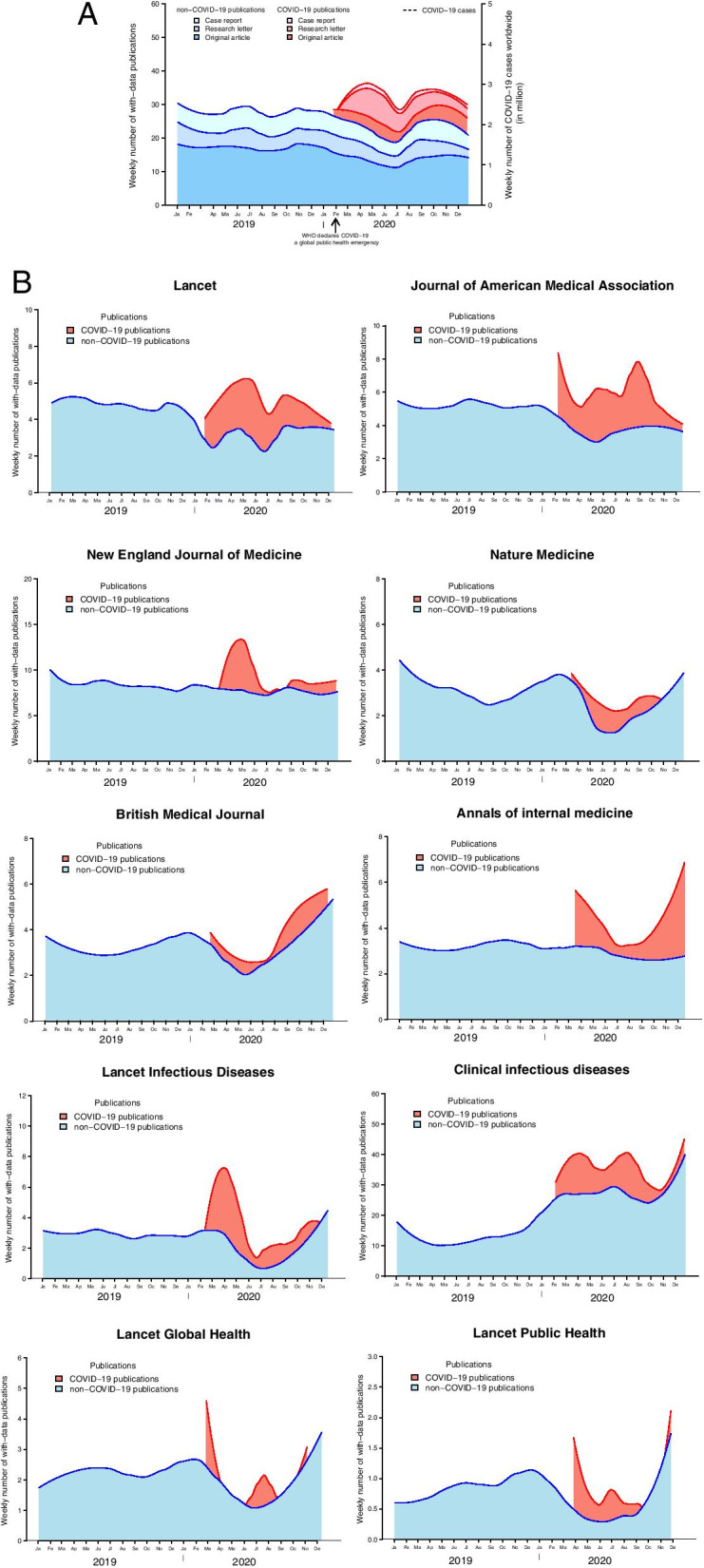
Weekly number of COVID-19 and non-COVID-19 publications with original data. These graphs show the publication dynamics in the journals included, from January 1st 2019 to January 1st 2021. We present in Panel **A** the top six general journals (New England Journal of Medicine, Lancet, Journal of American Medical Association, Nature Medicine, British Medical Journal, and Annals of Internal Medicine), given the distinct distribution in journals related to infectious diseases and public health. We present the distribution in all journals in supplementary Fig. 1. Panel **B** shows the distribution in each journal. **A**. Overall. **B**. Per Journal

In the general journals (gathering NEJM, Lancet, JAMA, Nature Medicine, BMJ and Annals of Internal Medicine), COVID-19 publications showed a significant increase starting in January 30th 2020, from a median number of 4.0 (IQR: 2.8–5.5) publications in February to 19.5 (IQR: 15.8–24.8) in April 2020 (*p* < 0.001) (Fig. 2A). After this peak, COVID-19 publications displayed stability with a weekly plateau of 10.0 (IQR: 6.0–14.0) publications until December (*p* = 0.045 in comparison with April).

Non-COVID-19 publications showed stability in 2019 with a median number of 28.0 (IQR: 25.0–33.0) publications between January and December 2019. In contrast, when the pandemic started, a decrease was observed, and reached a median number of 22.0 (IQR: 20.5–23.3) publications in June 2020 (*p* = 0.074). After this decrease, non-COVID-19 publications showed a slight increase with a weekly number of 26.0 (IQR: 23.5–30.5) publications until December 2020 (*p* = 0.149 in comparison with June).

We present in the supplementary Fig. 1 the publication dynamics for all journals.

#### Publication dynamics per journal

As shown in Fig. 2B, the Lancet, JAMA, BMJ, Nature Medicine, Lancet Global Health and Lancet Public Health journals significantly decreased their production of non-COVID-19 studies after starting to publish COVID-19 studies: their median, weekly number of non-COVID-19 publications was of 19.0 (IQR 15.5–22.5), and 15.0 (IQR 10.5–22.5) in 2019 and 2020 respectively (*p* = 0.002).

The NEJM, Annals of Internal Medicine and Lancet Infectious Disease journals maintained their production of non-COVID-19 publications during the pandemic while starting to publish COVID-19 studies: their median, weekly number of non-COVID-19 publications was of 15.0 (IQR 10.0–17.5), and 13.0 (IQR 10.0–17.0) in 2019 and 2020 respectively (*p* = 0.467).

The Clinical Infectious Disease journal presented with a distinct pattern, and increased its production of non-COVID-19 publications in 2020, while starting to additionally publish COVID-19 studies: its median, weekly number of non-COVID-19 publications was of 12.0 (IQR 10.5–13.5), and 28.5 (IQR 24.0–33.0) in 2019 and 2020 respectively (*p* < 0.001).

### Simulation of the number of unpublished non-COVID-19 studies

Based on the publication dynamics in the time period from January 1st 2019 to January 30th 2020, we extrapolated what the publication dynamics of non-COVID-19 studies could have been from February 1st 2020 to December 31st 2020 (see methods), if the pandemic had not occurred (supplementary Fig. 2). We removed the studies published in the Clinical Infectious Disease journal, given its very specific distribution that would bias the simulation.

Based on the simulation, we estimated that 1632 non-COVID-19 studies could have been published without the pandemic, in the nine selected journals, from February 1st 2020 to December 31st 2020. Since 1344 non-COVID-19 studies were published, this represents 288 unpublished non-COVID-19 studies, which thus corresponds to an estimated decrease of 18% in the production of non-COVID-19 research.

### Impact of the COVID-19 pandemic on the publication type

**Fig. 3:**
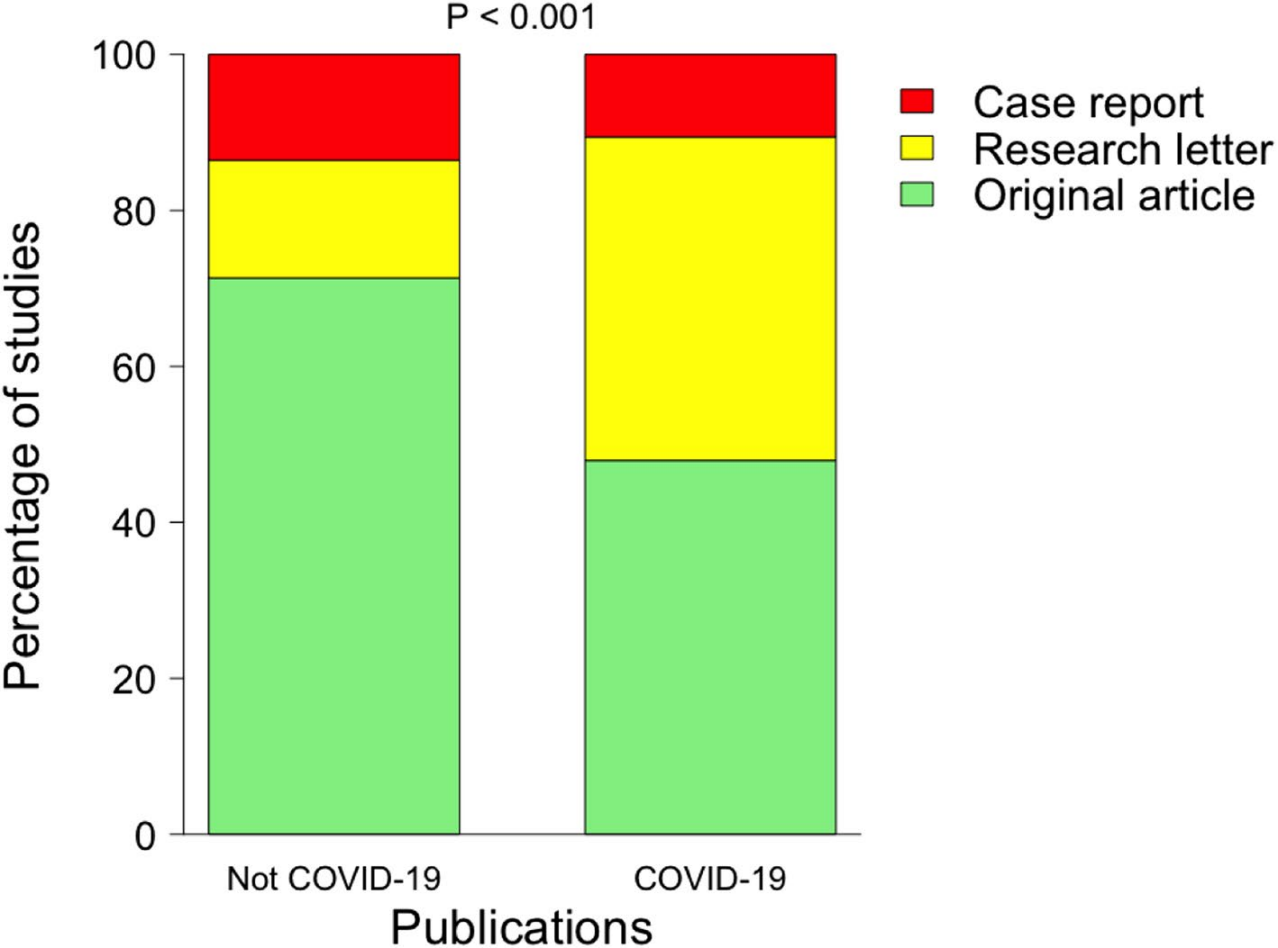
Publication type and COVID-19. This graph shows the distribution of the COVID-19 publications and non-COVID-19 publications, stratified per publication type (original articles, research letters, and case reports). A chi^2^ test was performed to assess the difference between the distributions. The distribution of the COVID-19 publications and non-COVID-19 publications, stratified per publication type in general journals is presented in supplementary Fig. 4

Among the 1022 COVID-19 publications, original articles, research letters and case reports accounted for 490 (47.9%), 424 (41.5%) and 108 (10.6%) publications respectively. Among the 5297 non-COVID-19 publications, original articles, research letters and case reports accounted for 3779 (71.3%), 800 (15.1%) and 718 (13.6%) publications respectively. (*P* < 0.001 for difference) (Fig. 3).

### COVID-19 pandemic and author multiplicity

**Fig. 4:**
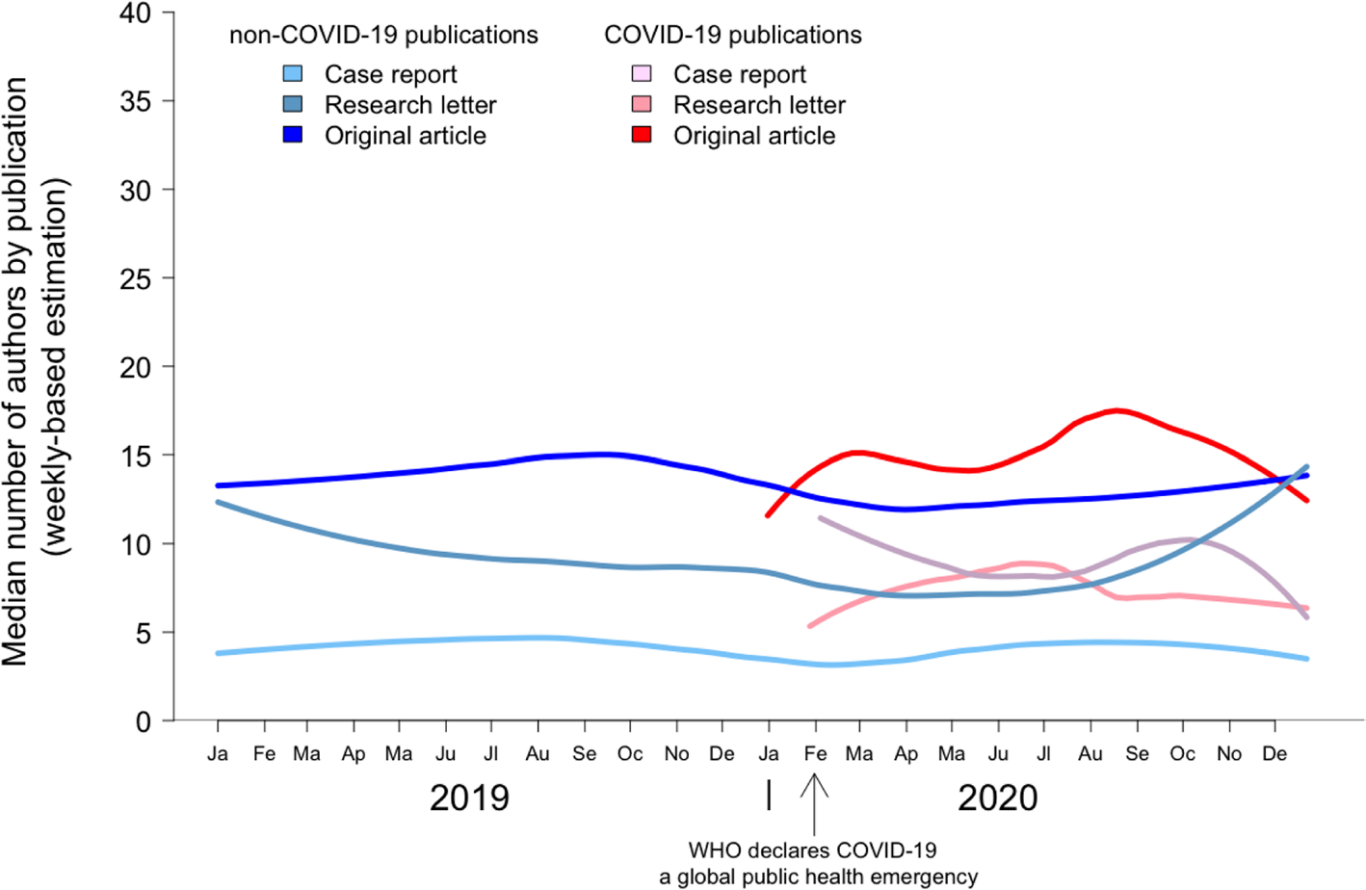
COVID-19 publications and the number of authors dynamics. This graph shows the dynamics of number of authors in COVID-19 publications and non-COVID-19 publications, stratified per publication type (original articles, research letters, case reports). The dynamics of number of authors in COVID-19 publications and non-COVID-19 publications, stratified per publication type, in general journals are presented in supplementary Fig. 5

**Fig. 5:**
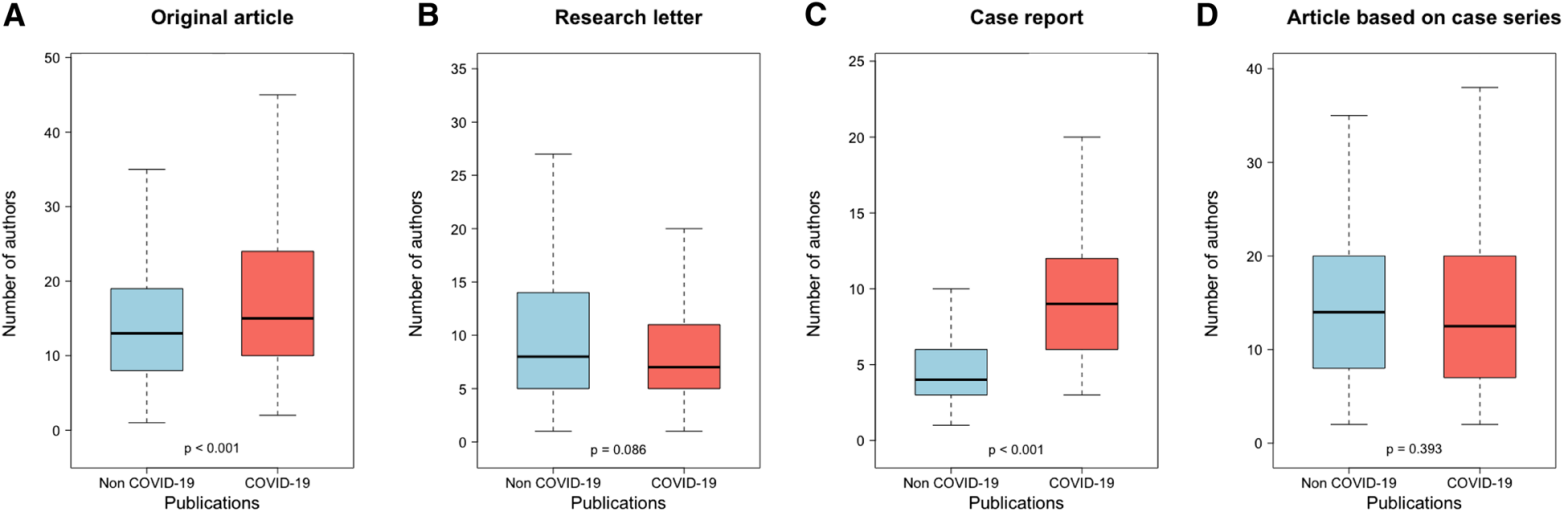
COVID-19 publications and author multiplicity. This graph shows the number of authors in COVID-19 publications and non-COVID-19 publications, stratified per publication type (original articles, research letters, and case reports). The article based on case series comprised original articles and research letters based on case series. A Wilcoxon test was performed to assess the difference between the distributions. The number of authors in COVID-19 publications and non-COVID-19 publications, stratified per publication type, in general journals is presented in the supplementary Fig. 6

The number of authors dynamics are presented in Fig. 4. For original articles, the median number of authors was 15.0 (IQR: 10.0–24.0) and 13.0 (IQR: 8.0–19.0) for COVID-19 and non-COVID-19 publications respectively (*p* < 0.001) (Fig. 5A). In research letters, the number of authors was 7.0 (IQR: 5.0–11.0) and 8.0 (IQR: 5.0–14.0) for COVID-19 and non-COVID-19 publications respectively (*p* = 0.086) (Fig. 5B). In case reports, the number of authors was 9.0 (IQR: 6.0–12.0) and 4.0 (IQR: 3.0–6.0) for COVID-19 and non-COVID-19 publications respectively (*p* < 0.001) (Fig. 5C). In original articles and research letters based on case series, the number of authors was 13.0 (IQR: 7.0–20.0) and 14.0 (IQR: 8.0–20.0) for COVID-19 and non-COVID-19 publications respectively (*p* = 0.393) (Fig. 5D).

To consider the influence that the number of patients in case report and case series may have on the number of authors, we calculated the ratio of the number of authors to the number of patients in case report and case series. In publications based on case report, the ratio was 6.0 (IQR: 3.0–10.0) and 4.0 (IQR: 3.0–5.0) for COVID-19 and non-COVID-19 publications respectively (*p* < 0.001) (supplementary Fig. 3A). In original articles and research letters based on case series, the ratio was 0.5 (IQR: 0.2–2.0) and 0.7 (IQR: 0.2–3.2) for COVID-19 and non-COVID-19 publications respectively (*p* = 0.057) (supplementary Fig. 3B).

## Discussion

In this meta-research gathering 22,525 publications in 10 major medical journals between 2019 and 2020, 6319 publications with original data (gathering original articles, research letters and case reports) were identified after reviewing. This study revealed the effects of the COVID-19 pandemic on the publication dynamics and the impact of COVID-19 research on non-COVID-19 research.

The exponential rise of COVID-19 publications began in February 2020, as the World Health Organization declared COVID-19 as a global public health emergency [24]. Only 4 months later, almost half of published medical research in top medical journals was dedicated to COVID-19, illustrating the commitment of editorial leadership to promote research related to the virus and provide time-sensitive data to scientifically address the problematics related to the pandemic [25, 26]. As a consequence, based on our simulation, there was a 18% decrease in the production of non-COVID-19 research, revealing to what extent the COVID-19 research has deeply impacted the non-COVID-19 research, a phenomenon highlighted and criticized by many researchers and key opinion leaders [6, 7, 9].

Overall, although most journals have created a specific section dedicated to the COVID-19 research [27, 28], they responded differently to the pandemic. Two main editorial strategies were identified based on the publication dynamics. First, we identified the journals that maintained the production of non-COVID-19 research while incorporating COVID-19 research. In these journals, as more published studies imply that editors worked on more studies, it results that they probably dedicate, in average, less time to conduct the editorial review. Therefore, it is possible that the scientific standards may have been, in some situations, considered with lower attention because of the need to provide timely scientific advances related to the pandemic. Second, we identified the journals that decreased the production of non-COVID-19 research while incorporating COVID-19 research, thus maintaining their overall production. In these journals, the editors rejected more papers than usual [29], supporting adherence and commitment to high scientific standards [13].

The number of authors significantly differed between COVID-19 and non-COVID-19 studies in original articles and case reports, which corroborates with a recent study by Zdravkoric et al. that focused on the top three medical journals [15]. This difference was especially noteworthy in case reports. Interestingly, when considering publications based on case series, there was no significant difference.

In addition, we showed a change in the publication type, that was mainly driven by the high proportion of research letters at the expense of original articles in COVID-19 publications, a phenomenon that has been previously illustrated [15] and might reflects how willingness to provoke immediate impact and provide novel insights could possibly have affected the quality of the medical research worldwide [30]. Given the current rapid change and adaptation in the medical research and resources [11, 31], such results would be of high interest for health researchers, public health officials and practitioners who are focused on controlling the pandemic while also sustaining the pace of non-COVID-19 research.

Nevertheless, these findings should be interpreted in the light of the efforts made around COVID-19, in particular the commitment around the estimation of COVID-19 cases and related deaths worldwide [1], the implementation of massive, international collaborations [4], the fast-tracking process of COVID-19 medical research [32], the vaccine development [33, 34], or the will to provide researchers with the most up to date information with, for instance, living systematic reviews on COVID-19 research [35–37]. Overall, innovations and discoveries have been brought and may help advancing medical research.

Although the worldwide population is progressively getting vaccinated, the COVID-19 pandemic still exerts a very harmful effect on many countries [38, 39]. In addition, the rise of many variants may challenge the efficacy of vaccines [40, 41] and delay the decrease in the number of cases and deaths. Accordingly, the medical research beyond COVID-19 is likely to be impacted in the long run. As such, we urge researchers to help continuing the evaluation on how health systems, medical research and resources are managed in pandemic time, as we attempted to accomplish in the present study.

### Limitations

Several limitations should be acknowledged. First, due to the very high number of studies, the references were not assessed by two independent reviewers. However, after the screening completion, three reviewers randomly checked 20% of the references for each reviewer, and a second screening was performed if more than 3% inconsistencies were observed. Second, for the same reason, we had to restrict the analyses on the highly cited medical journals only. This may have induced a selection bias, as the editorial strategies might be different in lower-impact journals. However high-impact journals likely reflect and drive the trends in publications and are therefore relevant examples to analyze the impact of the pandemic on medical research. Third, we investigated the impact of the COVID-19 pandemic on non-COVID-19 research by focusing on the publication dynamics, the publication type, and the phenomenon of author multiplicity. Critically appraising all studies would have been ideal and enhanced our demonstration; but this would have been a gigantic work that cannot be accomplished in such study design.

## Conclusion

To conclude, in this meta-research gathering original articles, research letters and case reports published in high-impact medical journals, we have shown the heterogeneity in the publication dynamics, and measured the impact of the COVID-19 pandemic in the production of non-COVID-19 studies. This study revealed how medical journals adapted to the pandemic, as some maintained the production of non-COVID-19 studies, and some decreased the production of non-COVID-19 studies. Last, we have identified an author multiplicity phenomenon in COVID-19 studies.

## Supplementary Information

**Additional file 1 **: **Methods:** Search strategy. **Figure 1**. Weekly number of COVID-19 and non-COVID-19 publications with original data (all journals). **Figure 2**. Simulation of the number of unpublished non-COVID-19 studies. **Figure 3** COVID-19 publications and the ratio of the number of authors to the number of patients. **Figure 4** Publication type and COVID-19 (general journals). **Figure 5** COVID-19 publications and the number of authors dynamics (general journals). **Figure 6** COVID-19 publications and multiplicity of authors (general journals).

## Data Availability

The data of the manuscript are available upon reasonable request. To obtain the data, please contact the corresponding author.

## Abbreviations

COVID: Coronavirus disease
PRISMA: Preferred Reporting Items for Systematic Reviews and Meta-Analyses
NEJM: New England Journal of Medicine
JAMA: Journal of American Medical Association
BMJ: British Medical Journal
NLP: Natural Language Processing
