# Supplementary material for: Impact of the COVID-19 pandemic on publication dynamics and non-COVID-19 research production
Source: BMC Med Res Methodol. 2021 Nov 22;21:255. doi: 10.1186/s12874-021-01404-9 (PMC8607966; doi:10.1186/s12874-021-01404-9)

**Impact of the COVID-19 pandemic on publication dynamics**

**and non-COVID-19 research production**

**Supplementary**

**METHODS**

Search strategy

**FIGURES**

Figure 1 - Weekly number of COVID-19 and non-COVID-19 publications with original data (all journals)

Figure 2 – Simulation of the number of unpublished non-COVID-19 studies

Figure 3 - COVID-19 publications and the ratio of the number of authors to the number of patients

Figure 4- Publication type and COVID-19 (general journals)

Figure 5- COVID-19 publications and the number of authors dynamics (general journals)

Figure 6- COVID-19 publications and multiplicity of authors (general journals)

**METHODS**

Search strategy

**Years:** from 2019-01-01 to 2021-01-01.

**Database:** PubMed

**Search terms:** A systematic literature search was performed in PubMed for articles published in NEJM, Lancet, JAMA, Nature Medicine, BMJ, Lancet Infectious Disease, Annals of Internal Medicine, Lancet Global Health, Lancet Public Health and Clinical Infectious Disease. The literature search was performed between January 1, 2019 and January 1, 2021. Two independent researchers (VG, KL) implemented the search strategy and retrieved the references. Any discrepancies were discussed by the two reviewers and resolved by consensus, or where necessary, through adjudication by a third reviewer. The following search string was used in PubMed: "The New England journal of medicine"[Journal] OR "Lancet"[Journal] OR "JAMA"[Journal] OR "JAMA internal medicine"[Journal] OR "Nature medicine"[Journal] OR "BMJ"[Journal] OR "Annals of internal medicine"[Journal] OR "The Lancet Infectious diseases"[Journal] OR " The Lancet. Global health"[Journal] OR "Lancet Public Health"[Journal] OR "PLoS medicine"[Journal] OR "Clin Infect Dis"[Journal] AND "2019/12/01"[Date - Publication]: "2020/05/20"[Date - Publication]. The following terms were added to the search for COVID-related articles: COVID-19 OR 2019-nCoV OR wuhan coronavirus OR China coronavirus OR novel coronavirus OR SARS-CoV-2 OR "severe acute respiratory syndrome coronavirus 2" OR COVID-19 OR coronavirus disease 2019 OR Novel Coronavirus Pneumonia.

The references of the included medical articles and relevant reviews were scanned for potentially relevant medical articles that may have been missed in the literature search. We also requested potentially eligible medical articles from content experts.

**FIGURES**

**Figure 1 - Weekly number of COVID-19 and non-COVID-19 publications with original data (all journals)**

This graph shows the publication dynamics in all the journals included, from January 1^st^ 2019 to January 1^st^ 2021.

**
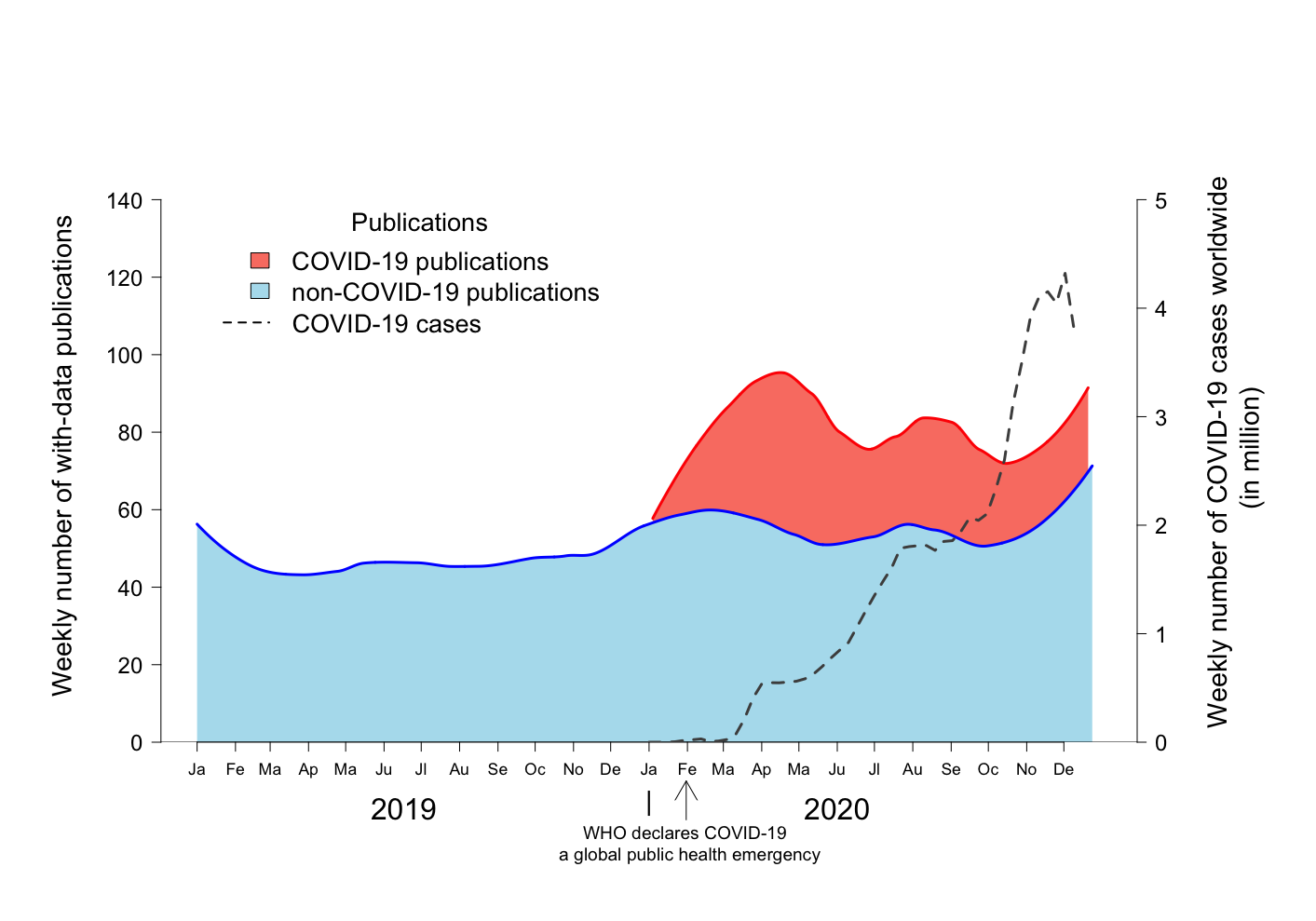
**

**Figure 2 – Simulation of the number of unpublished non-COVID-19 studies**

This graph shows how we extrapolate the publication dynamics of non-COVID-19 publications in 2020 based on the actual trends in 2020. Based on this approach, we estimated that an additional 288 non-COVID-19 studies could have published in 2020 if the pandemic did not occur.


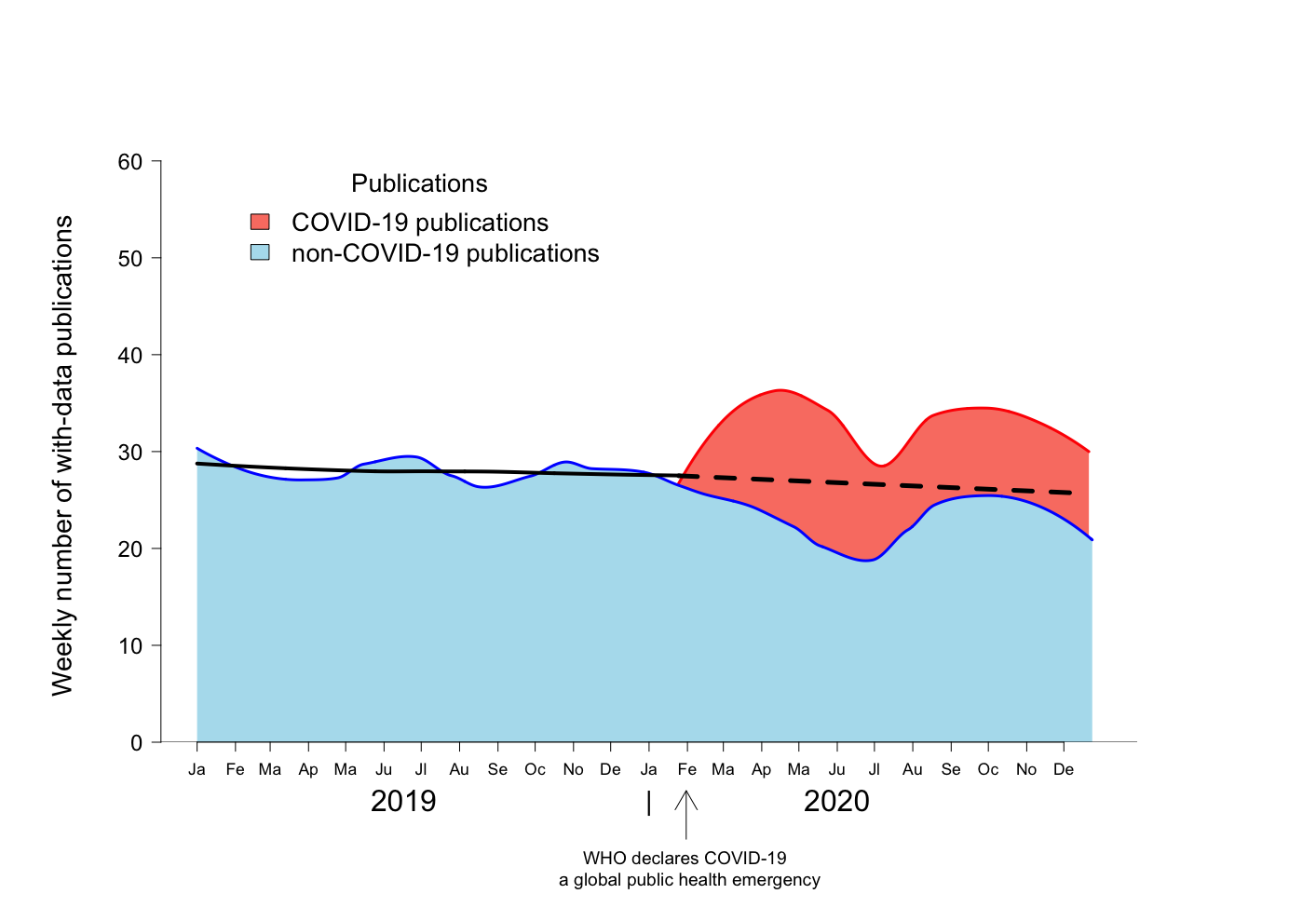


**Figure 3 - COVID-19 publications and the ratio of the number of authors to the number of patients**

This graph shows the ratio of the number of authors to the number of patients in case reports and in publications based in case series (excluding case report), in COVID-19 publications and non-COVID-19 publications. We first present the results in all journals included, and then in general journals.

*1. All journals*


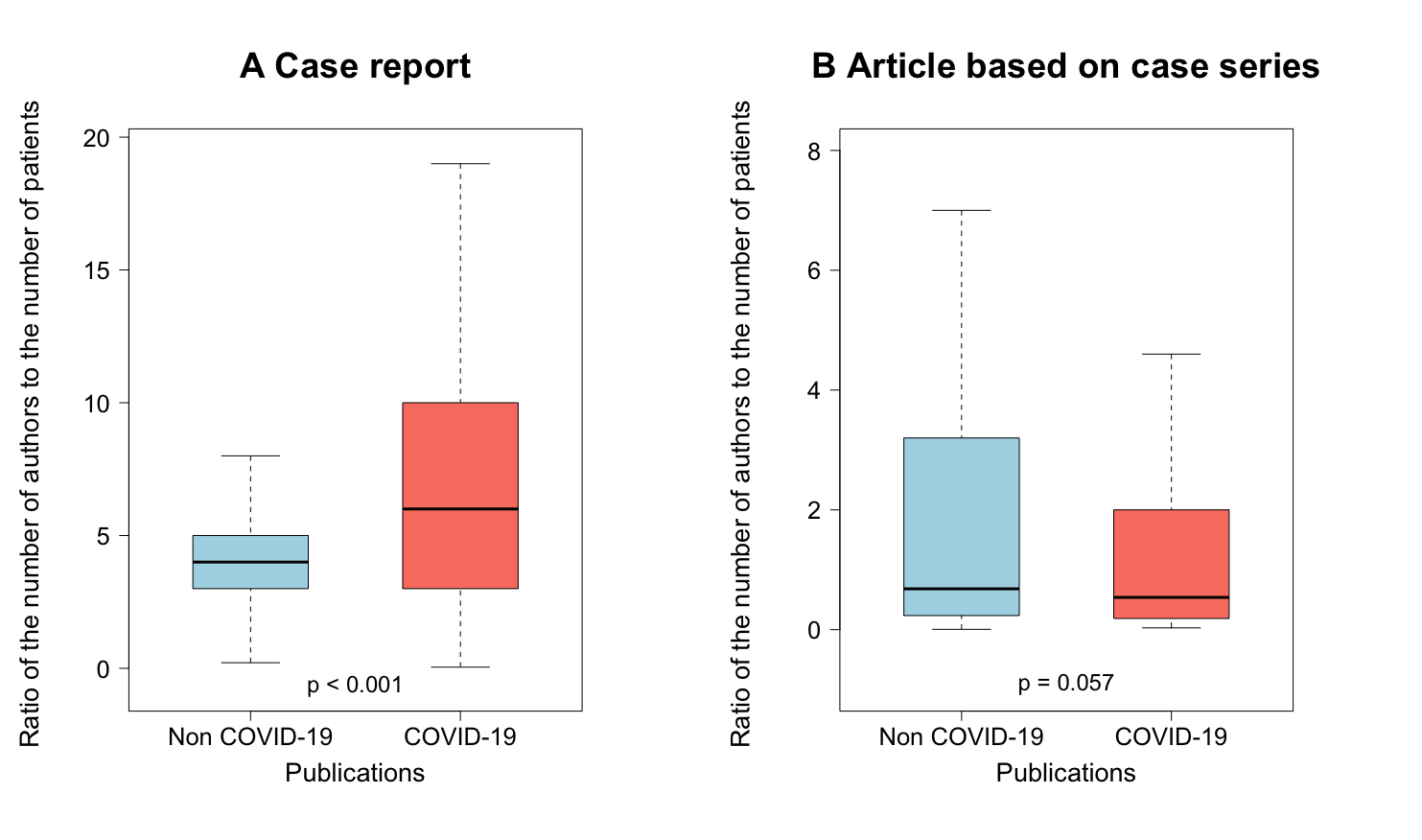


*2. General journals*


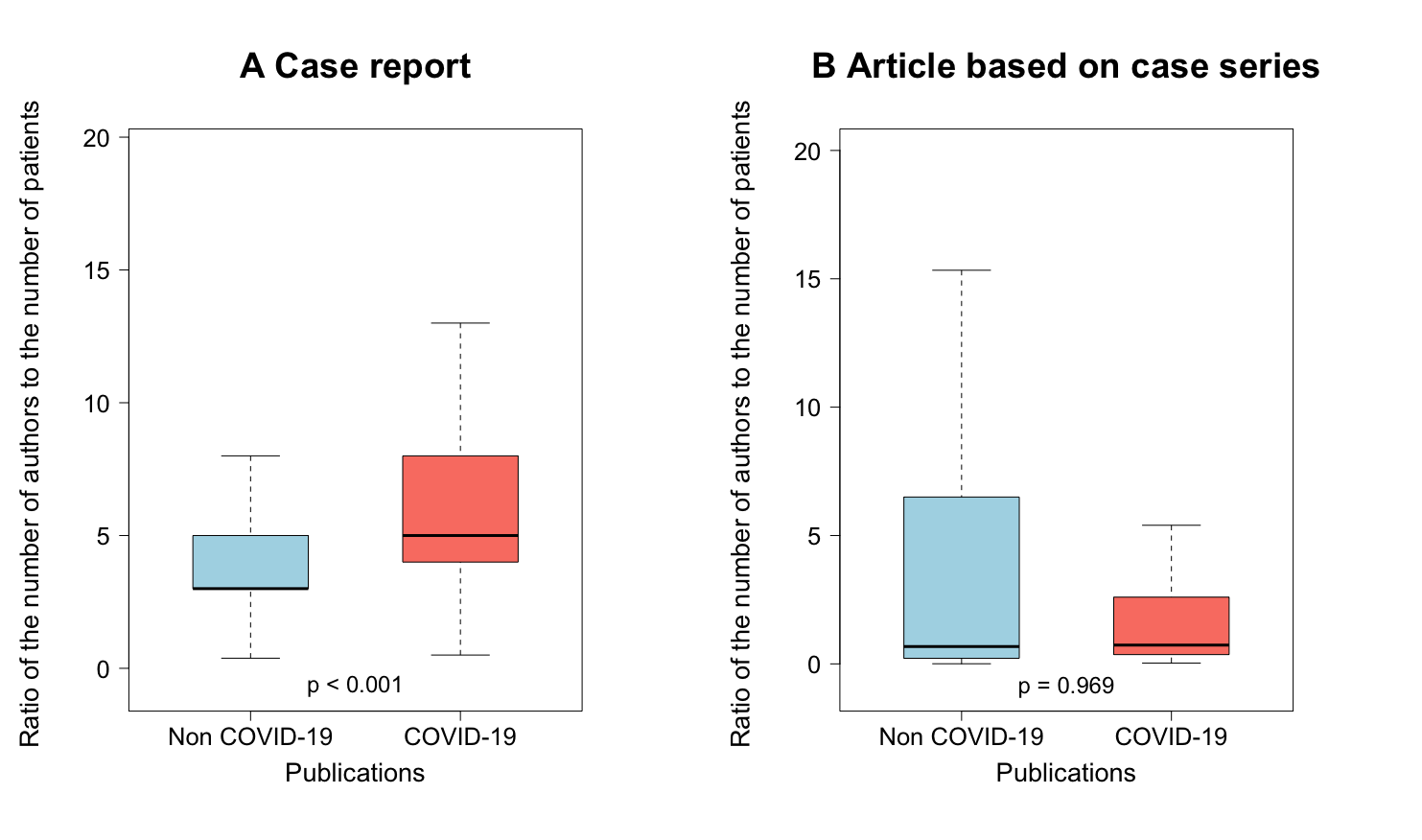


**Figure 4- Publication type and COVID-19 (general journals)**

This graph shows the distribution of the COVID-19 publications and non-COVID-19 publications, stratified per publication type (original articles, research letters, case reports, and without-data papers), in the general journals.


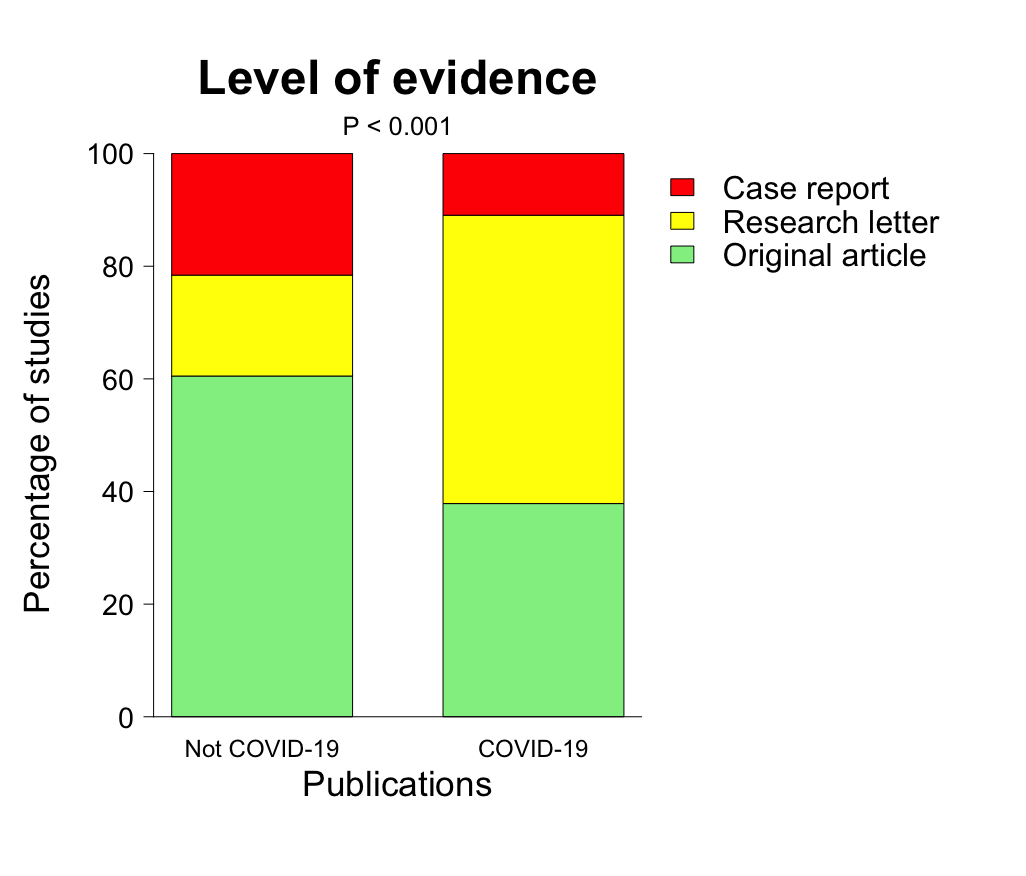


**Figure 5- COVID-19 publications and the number of authors dynamics (general journals)**

This graph shows the dynamics of number of authors in COVID-19 publications and non-COVID-19 publications, stratified per publication type (original articles, research letters, case reports, and without-data papers) in the general journals (NEJM, Lancet, JAMA, Nature Medicine, BMJ, Annals of internal medicine).


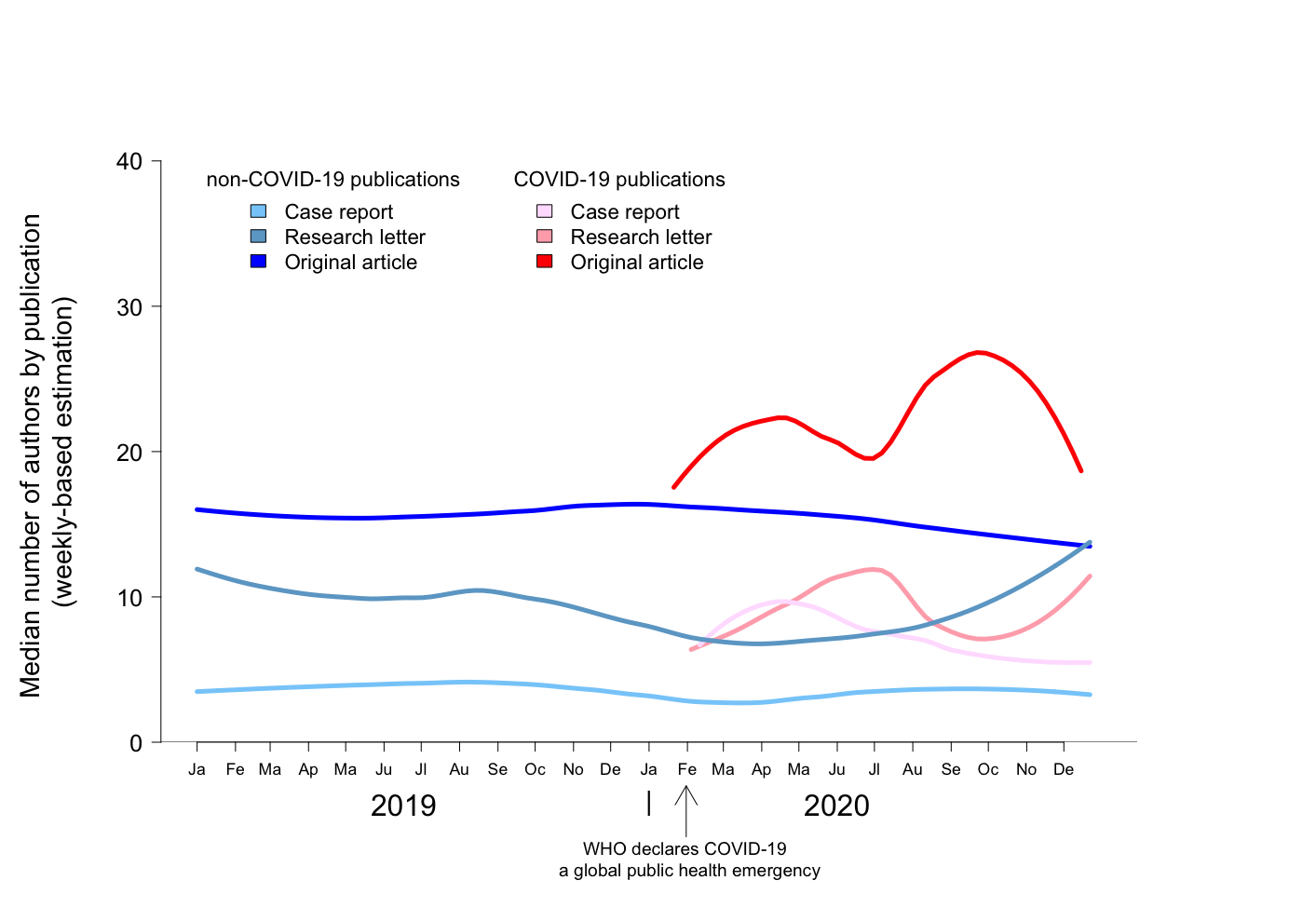


**Figure 6- COVID-19 publications and author multiplicity (general journals)**

This graph shows the number of authors in COVID-19 publications and non-COVID-19 publications, stratified per publication type (original articles, research letters, case reports, and without-data papers), in the general journals (NEJM, Lancet, JAMA, Nature Medicine, BMJ, Annals of internal medicine).


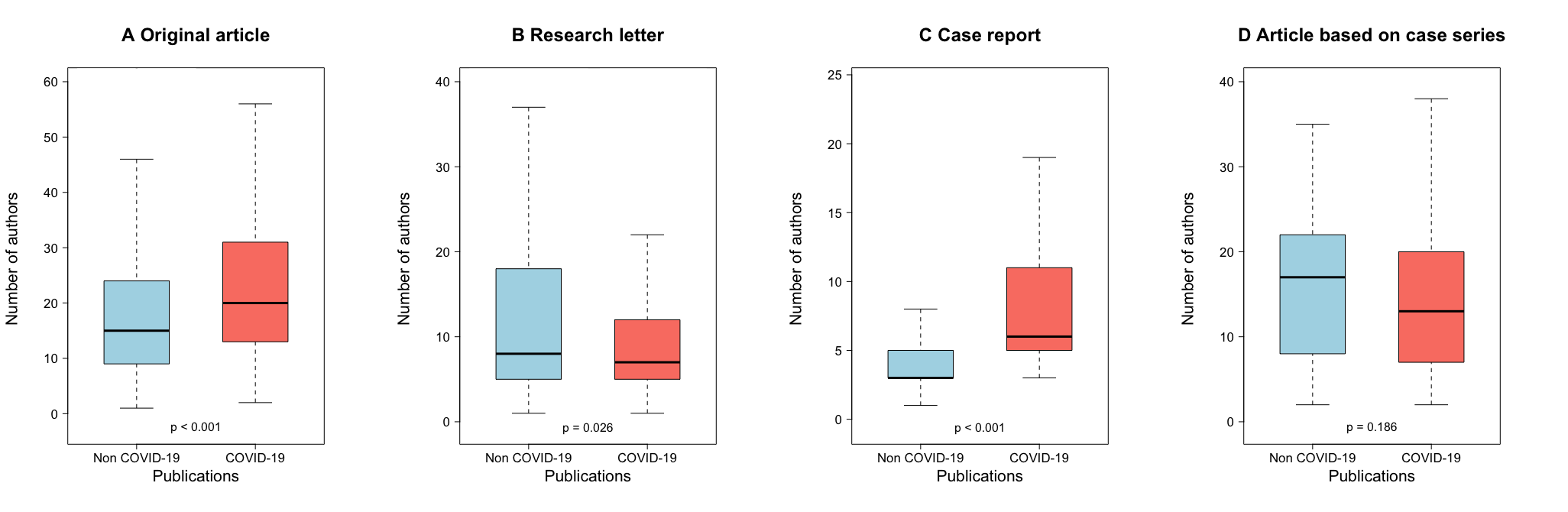

Supplement: Supplementary file 1 — Additional file 1 : Methods: Search strategy. Figure 1. Weekly number of COVID-19 and non-COVID-19 publications with original data (all journals). Figure 2. Simulation of the number of unpublished non-COVID-19 studies. Figure 3 COVID-19 publications and the ratio of the number of authors to the number of patients. Figure 4 Publication type and COVID-19 (general journals). Figure 5 COVID-19 publications and the number of authors dynamics (general journals). Figure 6 COVID-19 publications and multiplicity of authors (general journals). [file 12874_2021_1404_MOESM1_ESM.docx]
